# Purification, crystallization and preliminary crystallographic analysis of soybean mature glycinin A1bB2

**DOI:** 10.1107/S1744309113019684

**Published:** 2013-07-27

**Authors:** Krisna Prak, Bunzo Mikami, Takafumi Itoh, Takako Fukuda, Nobuyuki Maruyama, Shigeru Utsumi

**Affiliations:** a Laboratory of Food Quality Design and Development, Graduate School of Agriculture, Kyoto University, Gokasho, Uji, Kyoto 611-0011, Japan; b Laboratory for Molecular Cell Biology, Medical Research Council, University College London, London WC1E 6BT, England; c Laboratory of Applied Structural Biology, Division of Applied Life Sciences, Graduate School of Agriculture, Kyoto University, Gokasho, Uji, Kyoto 611-0011, Japan; d Division of Applied Biochemistry and Biomolecular Engineering, Department of Bioscience, Fukui Prefectural University, 4-1-1 Matsuoka Kenjyoujima, Eiheiji-cho, Yoshida-gun, Fukui 910-1195, Japan

**Keywords:** A1bB2, glycinin, soybean, homohexamer structure

## Abstract

Glycinin is one of the most abundant storage-protein molecules in soybean seeds and is composed of five subunits (A1aB1b, A1bB2, A2B1a, A3B4 and A5A4B3). A1bB2 was purified from a mutant soybean cultivar containing glycinin composed of only A5A4B3 and A1bB2. At 281 K the protein formed hexagonal, rectangular and rod-shaped crystals in the first [0.1 *M* imidazole pH 8.0, 0.2 *M* MgCl_2_, 35%(*v*/*v*) MPD], second [0.1 *M* sodium citrate pH 5.6, 0.2 *M* ammonium acetate, 30%(*v*/*v*) MPD] and third (0.1 *M* phosphate–citrate pH 4.2, 2.0 *M* ammonium sulfate) crystallization conditions, respectively. X-ray diffraction data were collected to resolutions of 1.85, 1.85 and 2.5 Å from crystals of the three different shapes. The crystals belonged to space groups *P*6_3_22, *P*2_1_ and *P*1, with unit-cell parameters *a* = *b* = 143.60, *c* = 84.54 Å, *a* = 114.54, *b* = 105.82, *c* = 116.67 Å, β = 94.99° and *a* = 94.45, *b* = 94.96, *c* = 100.66 Å, α = 107.02, β = 108.44, γ = 110.71°, respectively. One, six and six subunits of A1bB2 were estimated to be present in the respective asymmetric units. The three-dimensional structure of the A1bB2 hexamer is currently being determined.

## Abstract 2

Soybean mature glycinin was purified and crystallized and its preliminary crystallographic analysis is also reported.

## 1. Introduction   

Soybean (*Glycine max* L.) is one of the world’s leading economic crops; it has a high nutritional value and is the largest source of protein for human consumption and animal feed (Utsumi, 1992 ▶; Utsumi *et al.*, 1997 ▶). Owing to its high nutritional value and its beneficial effects, including the lowering of cholesterol (Kito *et al.*, 1993 ▶) and prevention of cancer, diabetes and obesity (Choudhary & Tran, 2011 ▶; Anderson *et al.*, 1995 ▶, 1999 ▶), consumption of soybean food products is increasing worldwide. On the other hand, soybean is believed to be a major cause of food-induced allergenic reactions. Seven soybean allergens are officially recognized by the Allergen Nomenclature Sub-Committee of the International Union of Immunological Societies (IUIS).

Soybean proteins are composed of two major components: β-­conglycinin (7S globulin) and glycinin (11S globulin). β-Conglycinin is composed of α (∼67 kDa), α′ (∼71 kDa) and β (∼50 kDa) subunits, whereas glycinin is composed of five subunits (Staswick *et al.*, 1984 ▶) divided into group I [A1aB1b (53.6 kDa), A1bB2 (52.2 kDa) and A2B1a (52.4 kDa)] and group II [A3B4 (55.4 kDa) and A5A4B3 (61.2 kDa)]. In developing seeds, the glycinins are synthesized as a single polypeptide precursor in the rough endoplasmic reticulum, where the co-translational signal peptide cleavage and subsequent trimer (proglycinin) assembly occur. The proteins are then transported and cleaved into an acidic (∼30 kDa) and a basic (∼20 kDa) polypeptide (except for A4 of A5A4B3) by a vacuolar processing enzyme in the protein-storage vacuole. The polypeptides are linked by a disulfide bond and the mature proteins assemble into hexamers (Dickinson *et al.*, 1989 ▶). The hexamers of glycinin are formed by random subunit combination. The proteins play different roles in food and non-food soybean protein products owing to their different physicochemical properties such as hydrophobicity, solubility, thermal stability and emulsification (Utsumi, 1992 ▶; Utsumi *et al.*, 1997 ▶). These properties are especially different between groups I and II of glycinin (Maruyama *et al.*, 2004 ▶; Prak *et al.*, 2005 ▶). A large number of European and Japanese soybean-allergenic patients have IgE antibodies to glycinin and β-conglycinin (Holzhauser *et al.*, 2009 ▶; Ito *et al.*, 2011 ▶). The structural analysis of these proteins is essential for the improvement of the nutritional qualities and functional properties of these proteins, as well as for elucidation of their allergenicity (Prak *et al.*, 2006 ▶, 2007 ▶; Prak & Utsumi, 2009 ▶; Tandang *et al.*, 2005 ▶). However, because of the heterogeneity of the molecular species, it is difficult to crystallize mature glycinin prepared from normal soybean cultivars (Utsumi, 1992 ▶). Using the *Escherichia coli* expression system, we can only obtain 11S globulin in the form of proglycinin. To obtain the mature glycinin structure, we need to prepare the protein from a mutant soybean cultivar; we thus successfully elucidated the structure of the glycinin A3B4 homohexamer (Adachi *et al.*, 2003 ▶).

In this study, we report the isolation, purification and crystallization of the A1bB2 mature glycinin subunit from a mutant soybean cultivar, as well as the X-ray diffraction results obtained.

## 2. Materials and methods   

### 2.1. Protein isolation and purification   

The glycinin A1bB2 homohexamer was isolated and purified from a mutant soybean cultivar containing glycinin composed of only A1bB2 and A5A4B3. The protein in 10 g defatted seed powder was extracted with 120 ml buffer *A* [30 m*M* Tris–HCl pH 8.0, 10 m*M* β-­mercaptoethanol (βME), 1 m*M* EDTA, 0.1 m*M* *p*-amidinophenylmethanesulfonyl fluoride hydrochloride (*p*-APMSF), 0.02%(*w*/*v*) NaN_3_, 0.2 m*M* pepstatin A, 0.5 µg ml^−1^ leupeptin] by stirring for 2 h at room temperature. The soluble and insoluble materials were separated by centrifugation at 24 000*g* for 30 min at 277 K. 0.98 g l^−1^ NaHSO_3_ was added to the supernatant and the pH of the extract was adjusted to pH 6.4 with HCl at 277 K. After centrifugation at 24 000*g* for 30 min at 277 K, the precipitate was dissolved in 60 ml buffer *B* [0.2 *M* HEPES pH 7.0, 0.4 *M* NaCl, 10 m*M* βME, 1 m*M* EDTA, 0.1 m*M* *p*-APMSF, 0.02%(*w*/*v*) NaN_3_]. Ammonium sulfate was added to the aliquot to 50% saturation and stirred for 15 min at room temperature before centrifugation at 24 000*g* for 30 min at 293 K. Ammonium sulfate was then added to the supernatant to 70% saturation and stirred for 30 min at room temperature before centrifugation at 24 000*g* for 30 min at 293 K. The protein in the precipitate was dissolved in 2 ml buffer *C* [0.2 *M* HEPES pH 7.0, 0.4 *M* NaCl, 10 m*M* βME, 1 m*M* EDTA, 0.02%(*w*/*v*) NaN_3_] and purified using a HiPrep 26/60 Sephacryl S-300 HR gel-filtration column (GE Healthcare) with buffer *C* as the mobile phase at a flow rate of 1 ml min^−1^. 1 µl protein samples from each fraction that was expected to contain A5A4B3 and A1bB2 were collected and analysed by 11%(*w*/*v*) SDS–PAGE under reducing conditions followed by N-terminal amino-acid sequencing analyses. The fractions containing A1bB2 were collected and subsequently purified using a HiLoad 26/10 Q Sepharose HP column (GE Healthcare). Elution was performed with a linear gradient from 0.2 to 0.5 *M* NaCl in buffer *C* without EDTA over a period of 150 min at a flow rate of 2 ml min^−1^. The fraction containing A1bB2 was concentrated to 10 mg ml^−1^ using a Vivaspin 20 with a 30 000 molecular-weight cutoff polyethersulfone membrane (Vivascience, Germany) and used for crystallization.

### 2.2. Crystallization   

Initial screening was performed by the sitting-drop vapour-diffusion method using a CrystalEX 96-well crystallization plate and the crystal screening kits Crystallization Basic Kit for Proteins, Crystallization Extension Kit for Proteins (Sigma) and Wizard Classic I and II (Emerald BioSystems). 1 µl protein sample (10 mg ml^−1^) in buffer *B* was mixed with 1 µl reservoir solution. Crystallization was performed at 281 and 293 K. After a few weeks several crystals appeared. Crystals grown in the first [0.1 *M* imidazole pH 8.0, 0.2 *M* MgCl_2_, 35%(*v*/*v*) MPD], second [0.1 *M* sodium citrate pH 5.6, 0.2 *M* ammonium acetate, 30%(*v*/*v*) MPD] and third (0.1 *M* phosphate–citrate pH 4.2, 2.0 *M* ammonium sulfate) crystallization conditions at 281 K were picked up in a loop and used for in-house diffraction data collection.

### 2.3. Diffraction data collection and processing   

A crystal grown in the third crystallization condition was soaked in 2.0 *M* ammonium sulfate, 0.1 *M* phosphate–citrate pH 4.2 containing 30%(*w*/*v*) MPD solution before flash-cooling and analysis of the diffraction images using an in-house Bruker HI-STAR detector coupled with a MAC Science M18XHF rotating-anode generator. The collected images were processed with *SADIE* and *SAINT* (Bruker). Crystals grown in the first and the second crystallization conditions were directly flash-cooled without cryoprotectant. These crystals were stored in liquid nitrogen after in-house diffraction checking and were used for X-ray diffraction data collection using ADSC Q315 and Rigaku JUPITER210 CCD detectors at 100 K on beamlines BL41XU and BL38B1 at SPring-8, Japan. The collected images were processed using *HKL*-2000 and *SCALEPACK* (Otwinowski & Minor, 1997 ▶). Cell-content analysis was performed with the *MATTHEWS_COEF* program in the *CCP*4 package (Winn *et al.*, 2011 ▶).

### 2.4. N-terminal amino-acid sequence analysis   

After the main protein bands had been excised from the SDS–PAGE gels, the proteins were extracted from the gel with SDS buffer [50 m*M* Tris–HCl pH 6.8, 2%(*w*/*v*) SDS, 10%(*v*/*v*) glycerol]. The extracted proteins were subjected to N-terminal amino-acid sequencing using a Procise 492 protein sequencer (Applied Bio­systems) after they had been blotted onto a PVDF membrane using a ProSorb cartridge (Applied Biosystems).

### 2.5. Confirmation of the expression of A1bB2 in mutant soybean   

Seeds of the soybean 11S globulin mutant were grown in pots and developing cotyledons were harvested for the preparation of total RNA according to Shirzadegan *et al.* (1991 ▶). The A1bB2 cDNA was amplified using the RNA LA PCR Kit (AMV) v.1.1 (Takara Bio). Initially, A1bB2 mRNA in total RNAs was reverse-transcribed by the primer 5′-CGC *GGATCC GGTACC CTGCAG GTCGAC* TTTTTTTTTTTTTTTTT-3′ that was composed of the region complementary to poly(A) and four restriction-enzyme sites (indicated in italics). PCR was performed for one cycle of 315 K for 20 min, 372 K for 5 min, 343 K for 15 min and 278 K for 5 min. The product was then used for PCR amplification of the cDNA using the primer 5′-TTCAGTTTCAGAGAGCAGCCACAGCAAAACGAGTCGCAG­ATCCAA­CG-3′ corresponding to the N-terminal sequence of A1bB2 and 5′-CGC*GGATCCGGTACCCTGCAGGTCGAC*TTTTTTTTTTTTTTTTT-3′. The reaction was performed using *LA Taq* DNA polymerase (Takara Bio) with 28 cycles of 367 K for 30 s, 333 K for 30 s and 345 K for 7 min. The amplified fragment with the expected size was blunted, phosphorylated and treated with *Bam*HI. The resultant fragment was then ligated with pET21d (Novagen) that had previously been treated with *Nco*I and blunted before treatment with *Bam*HI and dephosphorylation. The resulting plasmid was then transformed into DH5α. Insertion of the cDNA into the vector and the sequence of A1bB2 were confirmed by DNA sequence analysis using an ABI PRISM 3100 DNA analyzer (Applied Biosystems).

## 3. Results and discussion   

### 3.1. Protein purification   

**Figure 1:**
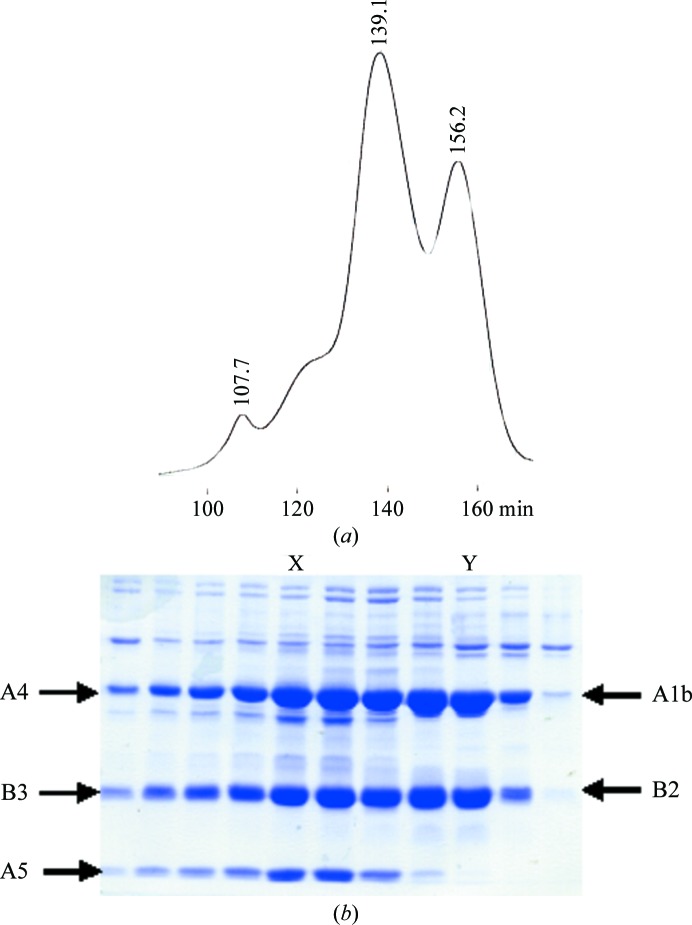
Purification of the soybean proteins. (*a*) Purification of the soybean glycinins using a HiPrep 26/60 Sephacryl S-300 HR gel-filtration column at a flow rate of 1 ml min^−1^. Each collected fraction contained 6 ml of the elution sample. (*b*) SDS–PAGE analysis under reducing conditions using 11%(*w*/*v*) gel and 1 µl of the elution samples at around 110–170 min. Arrows indicate the expected chains of soybean glycinin: A5, A4, A1b (acidic), B2 and B3 (basic) with molecular masses of 10.6, 30.1, 32.1, 20.5 and 20.7 kDa, respectively. X and Y indicate the fractions used for further N-terminal amino-acid sequence analysis of each protein band.

The glycinins were eluted from the gel-filtration column in two major peaks at 139.1 and 156.2 min (Fig. 1 ▶ *a*). SDS–PAGE analysis (Fig. 1 ▶ *b*) showed there were two different glycinin subunits. The major protein from the largest peak at 139.1 min was likely to be A5A4B3 (61.2 kDa) since it contained one basic and two acidic chains. The major protein in the second peak at 156.2 min was either A5A4B3 without the A5 acidic chain or was a different subunit of soybean glycinin composed of only one acidic and one basic chain. For confirmation, the corresponding band of the fractions indicated by X and Y in Fig. 1 ▶(*b*) was subjected to N-terminal amino-acid sequence analysis. It was found that the major protein eluted in the peak at 139.1 min was A5A4B3 glycinin corresponding to A5 (10.6 kDa), A4 (30.1 kDa) and B3 (20.7 kDa) and the major protein from the second peak at 156.2 min was A1bB2 corresponding to A1b (32.1 kDa) and B2 (20.5 kDa). For further confirmation, the mRNA and cDNA of the A1bB2 from the soybean cultivar were sequenced. The results showed an identical sequence to our previous A1bB2 gene (Prak *et al.*, 2005 ▶). It was similar to GMGY3 in GenBank except that base A at position 460 from the initiation codon of GMGY3 was replaced by C, but there was no change in the amino-acid sequence.

### 3.2. Diffraction data collection and processing   

**Figure 2:**
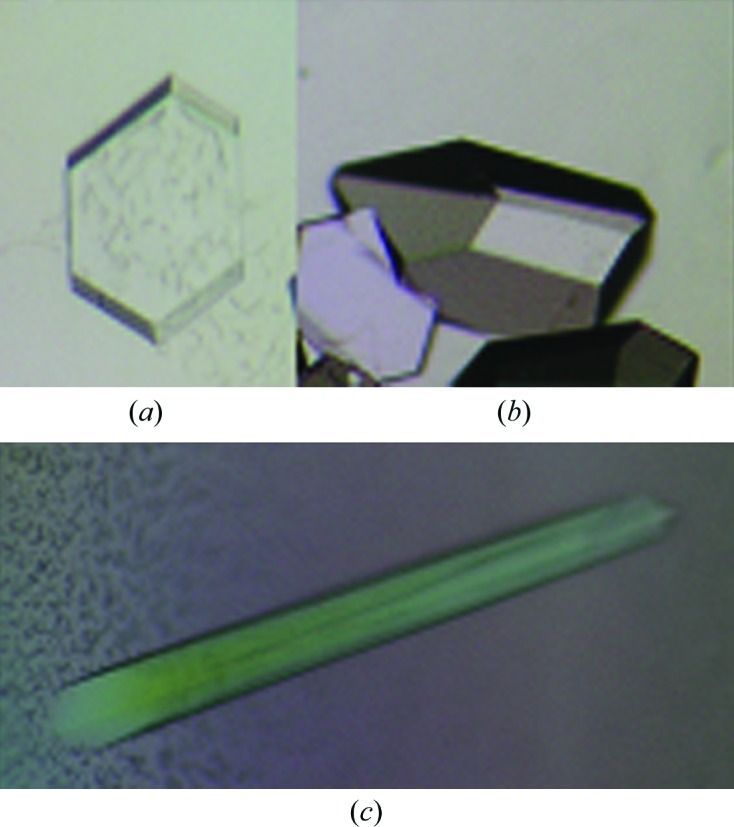
Crystals of soybean A1bB2 grown at 281 K in (*a*) 0.1 *M* imidazole pH 8.0, 0.2 *M* MgCl_2_, 35%(*v*/*v*) MPD (crystal type 1), (*b*) 0.1 *M* sodium citrate pH 5.6, 0.2 *M* ammonium acetate, 30%(*v*/*v*) MPD (crystal type 2) and (*c*) 0.1 *M* phosphate–citrate pH 4.2, 2.0 *M* ammonium sulfate (crystal type 3).

**Figure 3:**
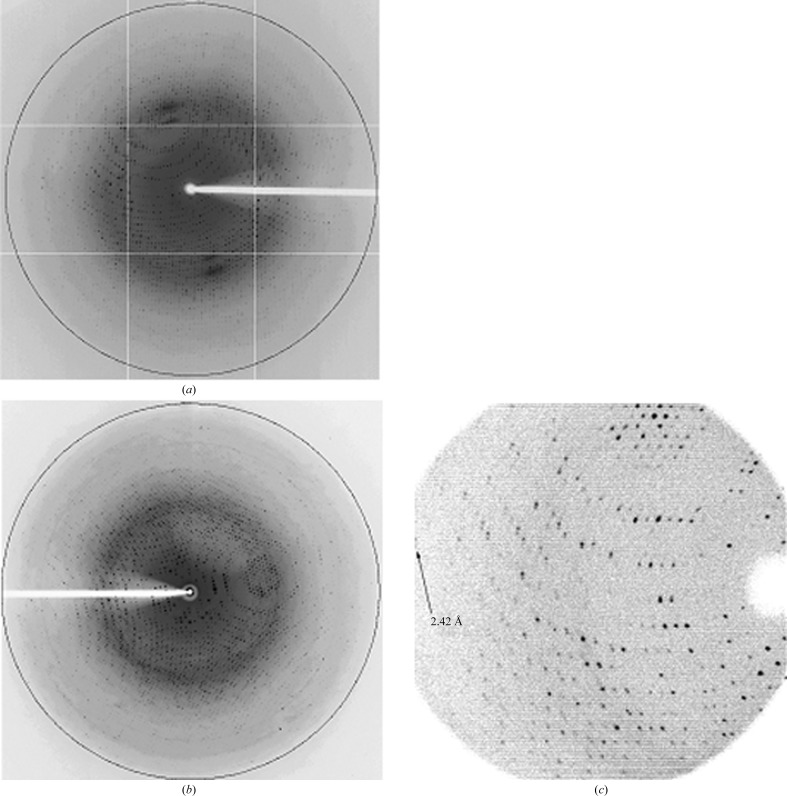
X-ray diffraction images of soybean A1bB2 glycinin crystals. (*a*) Diffraction image of crystal type 1, (*b*) diffraction image of crystal type 2 and (*c*) diffraction image of crystal type 3. The outer black circles in (*a*) and (*b*) correspond to 1.85 Å resolution.

A few weeks after crystallization setup at 281 K, crystals appeared. Hexagonal crystals appeared in the first crystallization condition consisting of 0.1 *M* imidazole pH 8.0, 0.2 *M* MgCl_2_, 35%(*v*/*v*) MPD (Fig. 2 ▶ *a*). A few rectangular crystals appeared in the second crystallization condition consisting of 0.1 *M* sodium citrate pH 5.6, 0.2 *M* ammonium acetate, 30%(*v*/*v*) MPD (Fig. 2 ▶ *b*) and one long rod-shaped crystal appeared in the third crystallization condition consisting of 0.1 *M* phosphate–citrate pH 4.2, 2.0 *M* ammonium sulfate (Fig. 2 ▶ *c*). A hexagonal crystal (crystal type 1) of dimensions of about 0.2 × 0. 15 × 0.05 mm, a rectangular crystal (crystal type 2) of dimensions of about 0.2 × 0.1 × 0.1 mm and a rod-shaped crystal (crystal type 3) of dimensions of about 0.5 × 0.05 × 0.05 mm were used for X-ray diffraction and data collection. Crystal types 1 and 2 diffracted to 1.85 Å resolution at SPring-8 (Figs. 3 ▶ *a* and 3 ▶ *b*). Crystal type 3 diffracted to 2.5 Å resolution on in-house X-ray analysis (Fig. 3 ▶ *c*). Data-collection and processing statistics are shown in Table 1 ▶. The crystals belonged to space groups *P*6_3_22, *P*2_1_ and *P*1, with unit-cell parameters *a* = *b* = 143.60, *c* = 84.54 Å, *a* = 114.54, *b* = 105.82, *c* = 116.67 Å, β = 94.99° and *a* = 94.45, *b* = 94.96, *c* = 100.66 Å, α = 107.02, β = 108.44, γ = 110.71°, respectively. The calculated Matthews coefficients (*V*_M_) were 2.41, 2.25 and 2.28 Å^3^ Da^−1^, with corresponding solvent contents of 0.49, 0.45 and 0.48, assuming the presence of one, six and six subunits in the asymmetric units, respectively (Matthews, 1968 ▶). In 129, 250 and 1392 frames, totals of 599 739, 1 443 947 and 125 424 reflections corresponding to 43 968, 235 443 and 87 040 unique reflections were collected with 99.4, 99.9 and 84.6% completeness and *R*_merge_ or *R*_sym_ values of 0.062, 0.059 and 0.083 to 1.85, 1.85 and 2.50 Å resolution for crystal types 1, 2 and 3, respectively. Research is in progress to determine the three-dimensional structure of A1bB2.

## Figures and Tables

**Table 1:** Data-collection statistics for the crystals of A1bB2 Values in parentheses are for the outermost resolution shell.

	Crystal type 1	Crystal type 2	Crystal type 3
No. of crystals	1	1	1
Beamline	BL41XU	BL38B1	In-house
Wavelength (Å)	1.0	1.0	1.5418
Detector	ADSC Q315 CCD	JUPITER210 CCD	Bruker HI-STAR
Crystal-to-detector distance (mm)	250.0	174.0	140.0
Rotation range per image (°)	1.0	1.2	0.25
Total rotation range (°)	129	300	348
Exposure time per image (s)	3.0	12	30
Resolution range (Å)	50–1.85 (1.92–1.85)	50–1.85 (1.92–1.85)	29.8–2.50 (2.60–2.50)
Space group	*P*6_3_22	*P*2_1_	*P*1
Unit-cell parameters (Å, °)	*a* = *b* = 143.60, *c* = 84.54	*a* = 114.54, *b* = 105.82, *c* = 116.67, β = 94.99	*a* = 94.45, *b* = 94.96, *c* = 100.66, α = 107.02, β = 108.44, γ = 110.71
Mosaicity (°)	0.275	0.281	
Total No. of measured reflections	599739	1443947	125424
Unique reflections	43968 (4294)	235443 (23314)	87040 (2802)
Multiplicity	13.6 (11.6)	6.1 (5.8)	1.4 (1.1)
〈*I*/σ(*I*)〉	44.2 (6.5)	34.5 (3.6)	14.3 (3.0)
Completeness (%)	99.4 (98.7)	99.9 (99.7)	90.8 (69.4)
*R*_merge_ † (%)	6.2 (34.5)	5.9 (42.0)	
*R*_sym_ ‡ (%)			8.3 (31.9)
Overall *B* factor from Wilson plot (Å^2^)	14.6	20.4	16.8

† *R*_merge_ = 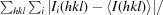 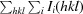.

‡ *R*_sym_ = 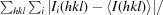 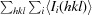.

## References

[bb1] Adachi, M., Kanamori, J., Masuda, T., Yagasaki, K., Kitamura, K., Mikami, B. & Utsumi, S. (2003). *Proc. Natl Acad. Sci. USA*, **100**, 7395–7400. 10.1073/pnas.0832158100 PMC165886 12771376

[bb2] Anderson, J. W., Johnstone, B. M. & Cook-Newell, M. E. (1995). *N. Engl. J. Med.* **333**, 276–282. 10.1056/NEJM199508033330502 7596371

[bb3] Anderson, J. W., Johnstone, B. M. & Cook-Newell, M. E. (1999). *Fed. Reg.* **64**, 57700–57733.

[bb4] Choudhary, S. P. & Tran, L. S. (2011). *Curr. Med. Chem.* **18**, 4557–4567. 10.2174/092986711797287593 21864283

[bb6] Dickinson, C. D., Hussein, E. H. & Nielsen, N. C. (1989). *Plant Cell*, **1**, 459–469. 10.1105/tpc.1.4.459 PMC159777 2562565

[bb8] Holzhauser, T., Wackermann, O., Ballmer-Weber, B. K., Bindslev-Jensen, C., Scibilia, J., Perono-Garoffo, L., Utsumi, S., Poulsen, L. K. & Vieths, S. (2009). *J. Allergy Clin. Immunol.* **123**, 452–458. 10.1016/j.jaci.2008.09.034 18996574

[bb9] Ito, K., Sjölander, S., Sato, S., Movérare, R., Tanaka, A., Söderström, L., Borres, M., Poorafshar, M. & Ebisawa, M. (2011). *J. Allergy Clin. Immunol.* **128**, 673–675. 10.1016/j.jaci.2011.04.025 21555150

[bb10] Kito, M., Moriyama, T., Kimura, Y. & Kambara, H. (1993). *Biosci. Biotechnol. Biochem.* **57**, 354–355. 10.1271/bbb.57.354 27314807

[bb11] Maruyama, N., Prak, K., Motoyama, S., Choi, S.-K., Yagasaki, K., Ishimoto, M. & Utsumi, S. (2004). *J. Agric. Food Chem.* **52**, 8197–8201. 10.1021/jf048786y 15612817

[bb30] Matthews, B. W. (1968). *J. Mol. Biol.* **33**, 491–497. 10.1016/0022-2836(68)90205-2 5700707

[bb12] Otwinowski, Z. & Minor, W. (1997). *Methods Enzymol.* **276**, 307–326. 10.1016/S0076-6879(97)76066-X 27754618

[bb13] Prak, K., Maruyama, Y., Maruyama, N. & Utsumi, S. (2006). *Peptides*, **27**, 1179–1186. 10.1016/j.peptides.2005.11.007 16356590

[bb14] Prak, K., Nakatani, K., Katsube-Tanaka, T., Adachi, M., Maruyama, N. & Utsumi, S. (2005). *J. Agric. Food Chem.* **53**, 3650–3657. 10.1021/jf047811x 15853415

[bb15] Prak, K., Nakatani, K., Maruyama, N. & Utsumi, S. (2007). *Protein Eng. Des. Sel.* **20**, 433–442. 10.1093/protein/gzm039 17720751

[bb16] Prak, K. & Utsumi, S. (2009). *J. Agric. Food Chem.* **57**, 3792–3799. 10.1021/jf8034258 19298043

[bb17] Shirzadegan, M., Christie, P. & Seemann, J. R. (1991). *Nucleic Acids Res.* **19**, 6055. 10.1093/nar/19.21.6055 PMC329073 1719489

[bb18] Staswick, P. E., Hermodson, M. A. & Nielsen, N. C. (1984). *J. Biol. Chem.* **259**, 13424–13430. 6541652

[bb19] Tandang, M. R., Atsuta, N., Maruyama, N., Adachi, M. & Utsumi, S. (2005). *J. Agric. Food Chem.* **53**, 8736–8744. 10.1021/jf050871y 16248579

[bb21] Utsumi, S. (1992). *Adv. Food Nutr. Res.* **36**, 89–208. 10.1016/s1043-4526(08)60105-9 1497851

[bb20] Utsumi, S., Matsumura, Y. & Mori, T. (1997). *Food Proteins and Their Applications*, edited by S. Damodaran & A. Paraf, pp. 257–291. New York: Dekker.

[bb22] Winn, M. D. *et al.* (2011). *Acta Cryst.* D**67**, 235–242.

